# Bioactivity and Molecular Docking Studies of Derivatives from Cinnamic and Benzoic Acids

**DOI:** 10.1155/2020/6345429

**Published:** 2020-05-21

**Authors:** Yunierkis Perez-Castillo, Tamires C. Lima, Alana R. Ferreira, Cecília R. Silva, Rosana S. Campos, João B. A. Neto, Hemerson I. F. Magalhães, Bruno C. Cavalcanti, Hélio V. N. Júnior, Damião P. de Sousa

**Affiliations:** ^1^Escuela de Ciencias Físicas y Matemáticas, Universidad de Las Américas, Quito, Ecuador; ^2^Department of Pharmacy, Federal University of Sergipe, CEP, 49100-000 São Cristóvão, Sergipe, Brazil; ^3^Department of Pharmaceutical Sciences, Federal University of Paraíba, CEP, 58051-970 João Pessoa, Paraíba, Brazil; ^4^Department of Clinical and Toxicological Analysis, School of Pharmacy, Laboratory for Bioprospection and Experiments in Yeast, LABEL, Federal University of Ceará, Fortaleza, CE, Brazil; ^5^Department of Physiology and Pharmacology, Federal University of Ceará, Fortaleza, CE, Brazil

## Abstract

Over the last decade, there has been a dramatic increase in the prevalence and gravity of systemic fungal diseases. This study aimed therefore at evaluating the antifungal potential of ester derivatives of benzoic and cinnamic acids from three *Candida* species. The compounds were prepared via Fischer esterification, and the antifungal assay was performed by the microdilution method in 96-well microplates for determining the minimal inhibitory concentrations (MICs). The findings of the antifungal tests revealed that the analogue compound methyl ferulate, methyl *o*-coumarate, and methyl biphenyl-3-carboxylate displayed an interesting antifungal activity against all *Candida* strains tested, with MIC values of 31.25-62.5, 62.5-125, and 62.5 *μ*g/ml, respectively. A preliminary Structure-Activity Relationship study of benzoic and cinnamic acid derivatives has led to the recognition of some important structural requirements for antifungal activity. The results of molecular docking indicate that the presence of the enoate moiety along with hydroxyl and one methoxy substitution in the phenyl ring has a positive effect on the bioactivity of compound 7 against *Candida albicans*. These observations further support the hypothesis that the antifungal activity of compound 7 could be due to its binding to multiple targets, specifically to QR, TS, and ST-PK. Additional experiments are required in the future to test this hypothesis and to propose novel compounds with improved antifungal activity.

## 1. Introduction

In the last decade, the number and severity of systemic fungal infections has increased intensely, and this has been linked specifically to an increased number of immunocompromised hosts, such as patients undergoing organ transplantation, receiving anticancer chemotherapy, suffering from AIDS, or in therapy with broad-spectrum antimicrobials [1, 2]. This factor, together with the limitations of available antifungal therapies and the crescent emergence of drug resistance, has created an urgent need for the development of new efficient broad-spectrum and safer antifungal agents, mainly with novel mechanisms for action [3, 4]*. Candida* species are broadly distributed in nature and constitute a part of the normal human microflora of the gastrointestinal tract, oral cavity, and vagina. However, a small percentage of the identified species is capable of causing opportunistic infections in humans, named as “candidiasis” [5–7]. Clinically, the most important pathogenic species of the *Candida* genus are *C. albicans*, *C. parapsilosis*, *C. krusei*, *C. tropicalis*, and *C. glabrata* [8].

Phenolic compounds comprise a broad class of secondary metabolites that possess a large spectrum of biological properties [9–12], and they have been successfully investigated for the treatment of systemic and superficial mycoses caused by different human pathogenic fungi [13–17]. Benzoic and cinnamic acids and their derivatives play an important role in antifungal activity, and several studies have focused on this bioactivity [18–25]. For example, Heleno and collaborators [26] reported the antifungal activity of the *p*-hydroxybenzoic and cinnamic acids as well as their protected glucuronide derivatives against different species of filamentous fungi, including species of the genus *Aspergillus*, *Penicillium*, and *Trichoderma.*

Thus, the objective of the present investigation was to investigate the antifungal potential of a set of 23 ester derivatives of benzoic and cinnamic acids from different *Candida* strains, including *C. albicans*, *C. krusei*, and *C. parapsilosis*. It also examined the Structure-Activity Relationship (SAR) of the screened compounds, thus, affording information for a better optimization of these classes of compounds as potential antimycotic agents.

## 2. Materials and Methods

### 2.1. General Procedure for the Synthesis of Compounds

A mixture of organic acid (0.5 g) and methanol (100 ml) was heated under reflux in the presence of sulfuric acid (0.8 ml) until the completion of the reaction which was checked by single spot TLC. Then, methanol was removed under reduced pressure and the solution was diluted with 20 ml of water. The product was extracted with ethyl acetate (30 ml). The organic phase was neutralized successively with NaHCO_3_ 5% and water, dried over anhydrous Na_2_SO_4_, and filtered. The ethyl acetate phase was separated, which on evaporation yielded the ester derivatives [27]. The structural identification of the products was made by infrared, ^1^H and ^13^C Nuclear Magnetic Resonance (NMR) analysis and comparison with the literature data [28–31].

### 2.2. In Vitro Antifungal Activity

Minimum inhibitory concentrations (MICs) for compounds benzoic and cinnamic derivatives were investigated by broth microdilution, according to the document M27-A3 [32], using RPMI broth (pH 7.0) buffered with 0.165 M MOPS (morpholinepropanesulfonic acid) (Sigma Chemical, St. Louis, MO). The compounds were dissolved in DMSO. To determine the susceptibility of the planktonic cells, drugs were tested at concentrations ranging from 0.98 to 1000 *μ*g/ml for the compounds benzoic and cinnamic derivatives. Yeasts and compounds were incubated in 96-well culture plates at 35°C for 24 h, and the results were examined visually, as recommended by CLSI [32]. The minimum inhibitory concentration (MIC) of each compound was determined as the concentration that inhibited 50% of fungal growth.

### 2.3. Molecular Docking

#### 2.3.1. Targets Selection

The selection of the potential targets of compound **7** was based on two criteria. First, there is evidence that coniferyl aldehyde derivatives can interfere with cell wall synthesis in *Candida albicans.* These previous results suggest that C-8 sterol isomerase (Uniprot ID A0A1D8PCB9, C-8-SI), Lanosterol 14-alpha demethylase (Uniprot ID P10613, L-14a-D), Thymidylate synthase (Uniprot ID P12461, TS), and Squalene monooxygenase (Uniprot ID Q92206, SQM) are potential targets of compounds bearing this scaffold. The second group of potential targets of compound 7 was selected based on a consensus target fishing approach. For this, the potential targets of the compound were predicted with five publicly available target fishing services: MolTarPred [33], SwissTargetPrediction [34], TargetNet [35], SuperPred [36], and Similarity Ensemble Approach (SEA) [37]. Any *Candida albicans* protein predicted as a potential target by any target fishing method was selected for modeling studies. In addition, the potential targets predicted by at least four methodologies were considered for homologs identification on *Candida albicans*. The latter consisted in the blast of the sequences of the predicted consensus targets against the *Candida albicans* proteome using the NCBI Blast server [38]. The predicted targets for which a homologous protein existed in *Candida albicans* were added to the list of potential targets of compound **7**. This second group of included enzymes: Carbonic anhydrase (Uniprot ID Q5AJ71, CA), Polyamine oxidase (Uniprot ID Q5AMQ8, PO), Quinone reductase (Uniprot ID Q59Z38, QR), and Serine/threonine protein kinase (Uniprot ID A0A1D8PH81, ST-PK).

#### 2.3.2. Molecular Docking

The X-ray structure of L-14a-D in complex with an inhibitor (PDB code 5tz1) and of CA (PDB code 6gwu) was obtained from the Protein Data Bank [39]. The rest of the potential targets had no structure determined, and homology models were obtained for them from the SWISS-MODEL server [40]. These models are searchable in the SWISS-MODEL repository using the above listed Uniprot IDs. The OMEGA software [41] was used to obtain the initial 3D structure of compound 7, and AM1-BCC charges were added to it with MOLCHARGE [42]. Molecular docking of compound 7 to its potential targets was performed with Gold [43] following the same procedure as in our previous research [44, 45]. Water molecules, cocrystalized ligands, and cofactors not involved in ligand binding were removed from the receptors. The receptor binding sites were defined based either on the cocrystalized ligands or functionally relevant residues. Primary docking was performed with the CHEMPLP scoring function, setting the Gold's search efficiency to 200%. In total, 30 different docking solutions were explored and all of them were rescored using the ChemScore, GoldScore, and ASP scoring functions. The most probable binding modes of compound 7 to the selected targets were selected according to *s* consensus scoring approach based on the scaling of each scoring function and their aggregation according to equation (1):

(1) $$\begin{array}{c} \;Z_{i}=\underset{j}{\sum }\frac{S_{i,j}-\bar{S_{j}}\;}{std\left(S_{j}\right)}. \end{array}$$

*S*_*i*,*j*_ is the score of conformer *i* according to scoring function *j* and $\bar{S_{j}}$ and *std*(*S*_*j*_) are the mean and standard deviation of the scoring function *S*_*j*_ across all conformers, respectively. Any ligand-receptor complex with an aggregated *Z* score higher than 1 was considered for further analysis.

#### 2.3.3. Molecular Dynamics and Free Energy of Binding Calculations

All molecular dynamics (MD) simulations and free energy of binding calculations were performed with Amber 2018 [14] following the procedure of our previous publication [45]. All complexes were subject to the same modeling procedure which included two minimization steps, the heating of the systems, their equilibration, production runs, the extraction of representative snapshots, and the estimation of the free energies of binding from them through MM-PBSA calculations. All MD simulations were performed in explicit solvents using the ff14SB and gaff force fields for proteins and non-amino acidic residues, respectively.

In brief, the antechamber was used to obtain the topology and force field modifications of the complexes. The systems were enclosed in a truncated octahedron box and solvated with TIP3P water molecules. Either Na^+^ or Cl^−^ ions were added to the solvated systems to neutralize any excess charge. Long-range electrostatic interactions were treated with the PME method with cutoff distances of 10 Å and 12 Å during the first minimization step and the remaining steps listed above, respectively.

The systems formed by the complexes, water, and counterions were first minimized during 500 steps of the steepest descent method followed by 500 cycles of conjugate gradient at constant volume with all atoms except water molecules and ions constrained with a force constant of 500 kcal/mol.Å^2^. During the second minimization step, no constraint was imposed on the system, and it took place for 1500 steps of the steepest descent method followed by 1000 cycles of conjugate gradient also at constant volume.

Next, the temperature of the systems was increased from 0 to 300 K at constant volume while keeping everything except water molecules and ions restrained with a force constant of 10 kcal/mol.Å^2^. Heating took place during 10000 steps with a time step of 2 fs. Once the systems reached 300 K in temperature, they were equilibrated at constant pressure (1 bar) and temperature (300 K). Isotropic position scaling with a relaxation time of 2 ps was used to control the pressure during equilibration.

The last snapshot from the equilibration stage was used as input to 20 MD simulations lasting 2 ns each. To ensure a better exploration of the conformational space of the systems, each of these simulations was initialized with different random velocities. During the heating, equilibration, and production run stages, the bonds involving hydrogen atoms were constrained with the SHAKE algorithm and the temperature was controlled using a Langevin thermostat with a collision frequency of 1.0 ps^−1^.

The MM-PBSA algorithm implemented in AmberTools 18 [46] was selected for the estimation of the free energies of binding of the ligand to the receptors. For these calculations, one MD snapshot every 200 ps was selected from every one of the previous production runs. This process was conducted by the selection of 200 diverse conformations of each system for MM-PBSA calculations. In addition, default implicit solvent parameters were used and the ionic strength was set to 100 mM.

## 3. Results and Discussion

### 3.1. Chemistry and Antifungal Activity of Compounds 1-23

**Figure 1:**
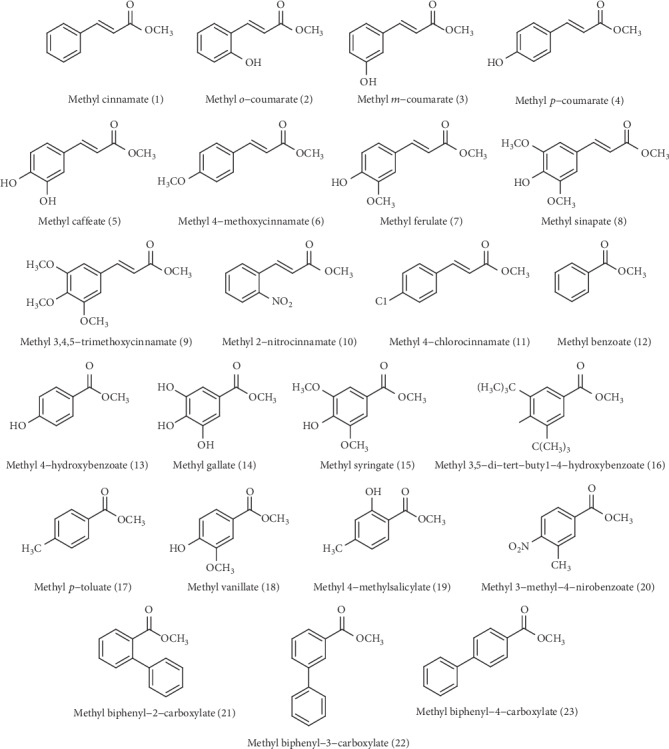
Chemical structures of the evaluated compounds.

In the present investigation, a series of 23 ester derivatives of benzoic and cinnamic acids (Figure 1) with different substitutions on the aromatic ring were assessed *in vitro* for their antifungal activity against three *Candida* species, namely, *C. albicans*, *C. krusei*, and *C. parapsilosis*. The antifungal assay was performed by the microdilution method in 96-well microplates for determining the minimal inhibitory concentrations (MICs). The results of the MIC testing, expressed in *μ*g/ml, are reported in Table 1. Among the 23 evaluated ester derivatives, the methyl ferulate (7) was found to exhibit the best antifungal activity, with MIC values of 62.5, 31.25, and 62.5 *μ*g/ml against *C. albicans*, *C. parapsilosis*, and *C. krusei*, respectively, followed by methyl biphenyl-3-carboxylate (22) (equal MIC = 62.5 *μ*g/ml) and methyl *o*-coumarate (2) (MIC = 62.5, 62.5, and 125 *μ*g/ml, respectively). Methyl 3,5-di-*tert*-butyl-4-hydroxybenzoate (16), methyl biphenyl-4-carboxylate (23), and methyl benzoate (12) were the less toxic compounds, with MIC values of 500 *μ*g/ml for the three *Candida* species. Furthermore, *C. parapsilosis* was the most susceptible species.

To obtain a better understanding of the relationship between the structural features of the screened ester derivatives and their antifungal effect, some structural and molecular characteristics which may contribute to the antifungal activity were recognized and will be discussed. In general, ester derivatives of the cinnamic acid were more efficient than their corresponding benzoic counterparts. For example, methyl ferulate (7) (MIC = 31.25 − 62.5 *μ*g/ml) had stronger antifungal activity than methyl vanillate (18) (MIC = 250 − 500 *μ*g/ml), methyl cinnamate (1) (equal MIC = 250 *μ*g/ml) was more bioactive than methyl benzoate (12) (equal MIC = 500 *μ*g/ml), methyl *p*-coumarate (4) (MIC = 125 − 250 *μ*g/ml) was more potent than methyl 4-hydroxybenzoate (13) (equal MIC = 250 *μ*g/ml), and methyl sinapate (8) (MIC = 125 − 250 *μ*g/ml) was more effective than methyl syringate (15) (MIC = 250 − 500 *μ*g/ml).

The addition of a hydroxyl group to the aromatic ring, in general, resulted in higher antifungal action. For example, when the bioactivity of the compounds methyl cinnamate (1), methyl *o*-coumarate (2), methyl *m*-coumarate (3), and methyl *p*-coumarate (4) was compared, it was found an increase in potency in the following order: 2 > 4 > 3 = 1 (MIC = 62.5 − 125, 125-250, 250, and 250 *μ*g/ml, respectively). A similar comparative trend can be seen when the compounds methyl benzoate (12) and methyl 4-hydroxybenzoate (13) were compared, once that 13 (equal MIC = 250 *μ*g/ml) was more bioactive than 12 (equal MIC = 500 *μ*g/ml). Furthermore, the position of the hydroxyl group on the benzene ring changes the activity, because compound 2 (*ortho*-hydroxyl; MIC = 62.5 − 125 *μ*g/ml) was more active than 3 (*meta*-hydroxyl; equal MIC = 250 *μ*g/ml) and 4 (*para*-hydroxyl; MIC = 125 − 250 *μ*g/ml). A study performed by Kim et al. (2011) [47] demonstrated that various benzaldehydes, like 2-hydroxy-3-methoxybenzaldehyde and 2-hydroxy-5-methoxybenzaldehyde, have been recognized to have a potent antifungal activity against different filamentous fungi, and a structure-activity relationship analysis showed that the antifungal effect was improved by the presence of an *ortho*-hydroxyl group on the aromatic ring.

However, the introduction of a second or third hydroxyl group into the benzene ring decreased the antifungal effect. For example, the compound methyl caffeate (5) (equal MIC = 250 *μ*g/ml) was less bioactive than methyl *p*-coumarate (4) (MIC = 125 − 250 *μ*g/ml), and methyl gallate (14) (MIC = 250 − 500 *μ*g/ml) was less potent than methyl 4-hydroxybenzoate (13) (equal MIC = 250 *μ*g/ml). Ambobaré and coworkers (2002) [19] reported that the introduction of a supplementary hydroxyl group in different positions on the aromatic ring of the benzoic acid derivatives strongly decreased the antifungal activity against *Eutypa lata* compared to salicylic acid, while the addition of two hydroxyl groups did not noticeably enhance the effectiveness. Furthermore, 2-hydroxyl group was the best replacement for the benzene ring. In another study, Sánchez-Maldonado and collaborators (2011) [48] evaluated the antimicrobial effect of twelve hydroxybenzoic and hydroxycinnamic acids against four gram-positive bacteria: *Lactobacillus plantarum*, *L. hammesii*, *Escherichia coli*, and *Bacillus subtilis*. These authors described how the activity of the hydroxybenzoic acids diminished significantly with an increasing number of hydroxyl groups. For example, the antibacterial activity of gallic acid (three hydroxyl groups on the aromatic ring) was about 2-10-fold lesser when compared with other hydroxybenzoic acids.

The presence a 5-hydroxyl group together with an additional methoxy group at the 3-position on *trans*-cinnamic acid methyl moiety appears to be important to enhance the antifungal activity, because the methyl ferulate (7; MIC = 31.25 − 62.5 *μ*g/ml) was more potent than the compounds 1 (methyl cinnamate, equal MIC = 250 *μ*g/ml), 4 (methyl *p*-coumarate, MIC = 125 − 250 *μ*g/ml), 5 (methyl caffeate, equal MIC = 250 *μ*g/ml), and 6 (methyl 4-methoxycinnamate, equal MIC = 250 *μ*g/ml). Furthermore, the increase of methoxy groups on the benzene ring remarkably decreased the antifungal effect, because compound 7 (one methoxy group; MIC = 31.25 − 62.5 *μ*g/ml) was more potent than 8 (two methoxy groups; MIC = 125 − 250 *μ*g/ml), and this was more active than 9 (three methoxy groups; MIC = 250 − 500 *μ*g/ml).With regard to the aromatic substitution within class benzoic, it was found that the increase of methoxy groups on the benzene do not affect the antifungal effect, once the compounds methyl gallate (14), methyl syringate (15), and methyl vanillate (18) exhibited the same activity (MIC = 250 − 500 *μ*g/ml). In contrast to the results reported herein, Sánchez-Maldonado and coworkers (2011) [48] observed that the antibacterial activity of hydroxybenzoic acids was enhanced through the replacement of a hydroxyl group by a methoxy group. Moreover, the presence of methoxy groups did not significantly influence the antimicrobial effect of cinnamic acids.

The introduction of bulky alkyl substituents into the aromatic ring resulted in a decrease in the antifungal effect. For example, the compound methyl 3,5-di-*tert*-butyl-4-hydroxybenzoate (16; two *tert*-butyl groups) was less potent (equal MIC = 500 *μ*g/ml) than compounds 14 and 15 (equal MIC = 250 − 500 *μ*g/ml). This diminution in the antifungal effect probably occurs due to steric hindrance caused by the presence of two *tert*-butyl groups. Similar results were also observed in others studies [49, 50].

The addition of a NO_2_ group at the 2-position of the methyl cinnamate, resulting in compound 10 (methyl 2-nitrocinnamate), did not increase antifungal activity, as compounds 1 and 10 presented the same antifungal potency (equal MIC = 250 *μ*g/ml). Furthermore, the compound methyl 3-methyl-4-nitrobenzoate (20; MIC = 250 − 500 *μ*g/ml) was less bioactive than the compound methyl *p*-toluate (17; MIC = 125 − 250 *μ*g/ml), and the compound methyl 4-chlorocinnamate (11; MIC = 250 − 500 *μ*g/ml) was less potent than the compound methyl cinnamate (1; equal MIC = 250 *μ*g/ml). These observations suggest that the decrease in electron diversity caused by electron-withdrawing groups (such as nitro or chlorine) resulted in a reduction of the antifungal effect. Likewise, compounds possessing electron donating groups (like methyl) exhibited better antifungal activity, as methyl *p*-toluate (17; MIC = 125 − 250 *μ*g/ml) was more active than its corresponding methyl cinnamate (1; equal MIC = 250 *μ*g/ml). Similar results were also obtained by Fu and collaborators (2010) [51]. These authors found that caffeic acid anilides with electron donating groups (such as methyl or methoxy) in *p*-position of the benzene ring displayed better antibacterial activity than those with electron-withdrawing groups (like bromide, nitro, or fluoride).

Lipophilicity has been extensively studied and is considered as one of the most important parameters influencing the antifungal activity [21, 23, 34]; however, no correlation was observed in this set of tested compounds, as most lipophilic compounds, like 3,5-di-*tert*-butyl-4-hydroxybenzoate (16) and methyl biphenyl-4-carboxylate (23), exhibited weak antifungal effect.

### 3.2. Molecular Docking

The molecular docking was performed with the most potent compound of the series, methyl ferulate (7). Otherwise noted in the Materials and Methods section, default parameters were used for all calculations described from here on. Compound 7 was docked at the binding sites of C-8-SI, L-14a-D, TS, SQM, CA, PO, QR, and ST-PK following the procedure described above. The results of the docking studies are presented in Table 2. For SQM, Gold could not find any valid docking solution and hence this target was excluded from any further analysis. The docking to the rest of the potential targets reveals that for most of them more than one possible binding mode of compound 7 is predicted. Overall, the potential complexes summarized in Table 2 show favorable interactions between the ligand and the receptor. These interactions include contacts with functionally important residues in the receptors. In addition, the best docking scores are predicted for L-14a-D, PO, and ST-PK.

Despite the meaningful complexes predicted by molecular docking, this type of modeling tool is primarily optimized for the processing of large databases of chemical compounds in a short time. For this aim, molecular docking uses simplified models that take into account a limited number of factors associated with molecular recognition. In consequence, the docking predicted complexes are not necessarily stable. Previous studies have shown that many predicted ligand-receptor complexes, although presenting favorable interactions might not be feasible [44, 45]. To further refine the possible mechanism of action of compound 7, MD simulations and MM-PBSA calculations were performed as described in the Materials and Methods section. The results of the estimation of the free energy of binding of compound 7 in the complexes listed in Table 2 are summarized in Table 3.

**Figure 2:**
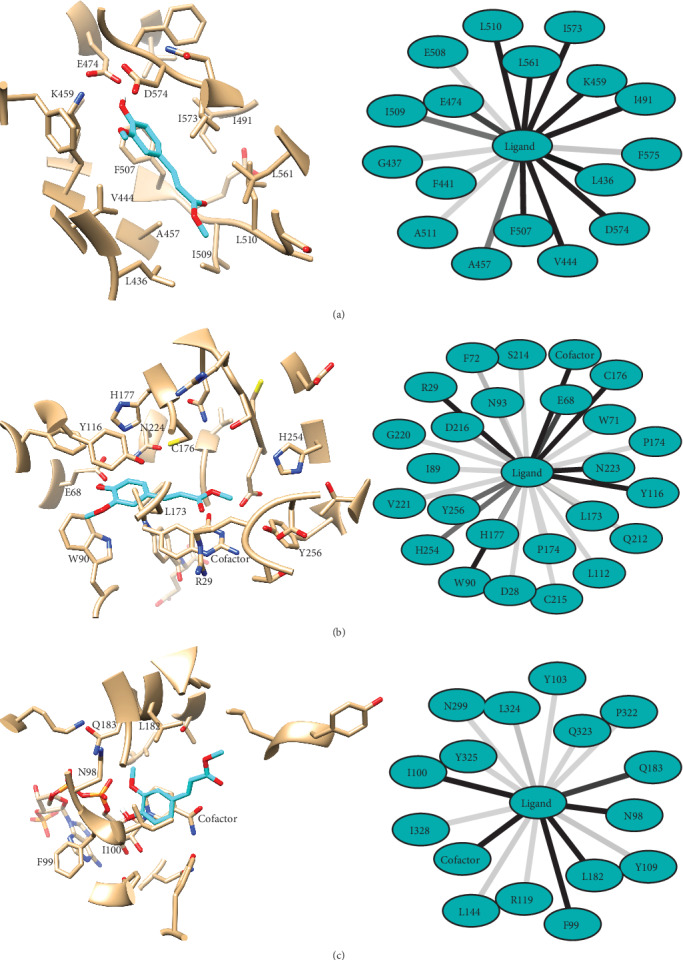
Predicted binding modes of compound 7 to ST-PK (a), TS (b), and QR (c) of *Candida albicans* (left). Compound 7 is depicted cyan and the receptors in gray, with noncarbon atoms following the scheme: blue for N, red for O, yellow for S, white for H, and orange for P. On the right side of the figure are represented the predicted interaction frequencies with the residues at the receptors binding sites. Darker lines indicate the higher frequencies of interaction. Only residues interacting with the ligand in more than 50% of the analyzed MD snapshots are labelled in the complexes' structures.

As seen in Table 3, compound 7 is predicted to bind stably to ST-PK, TS, and QR. According to our results, the lowest free energy of binding is obtained for ST-PK, followed by TS and QR. Supporting the previous observation, despite having the highest docking scores, the complexes predicted with L-14a-D are the less stable ones among all studied systems. The most probable binding mode of compound 7 to ST-PK, TS, and QR as well as the network of interactions that it forms with the receptors are presented in Figure 2. The most probable binding mode of the compound to each receptor was selected as that having the lowest predicted *Δ*G of binding.

The detailed description of the interactions observed between compound 7 and the ST-PK, TS, and QR receptors is discussed next. Figures of the predicted complexes of compound 7 with the receptors discarded due to its predicted poor *Δ*G of binding (L-14a-D, C-8-SI, PO, and CA) as well as their description are provided as Supporting Information. It must be considered that biomolecular complexes are dynamic systems, thus, the following analyses focus on the ligand-receptor interactions observed in more than 50% of the analyzed MD snapshots. In all cases, the complexes used for depiction are the centroids of the most populated clusters obtained from the 200 MD snapshots employed for the MM-PBSA calculations in each system.

Compound 7 is predicted to interact with QR through direct contact with the NADPH cofactor in all MD snapshots extracted for MM-PBSA calculations. This predicted binding mode would prevent the access of the enzyme's substrate to its binding site and conduct of the blockage of the enzyme functioning. Specifically, compound 7 is predicted to hydrogen bond, one of the phosphate groups of NADPH in 84% of the selected snapshots through its hydroxyl group. Less frequent hydrogen bonds are also predicted with the backbones of I100 and Y325 in 28% and 12.5% of the snapshots, respectively. In addition, the complex is stabilized by the stacking of the ligand's phenyl ring in front of the cofactor's nicotinamide group. Compound 7 also contacts N98, F99, I100, L182, and Q183 in most of the selected MD snapshots. All of these residues are located close to the cofactor and stabilize the orientation of the aromatic moiety of the compound in the receptor-binding pocket. On the other hand, the enoate moiety of the ligand shows low frequency of interaction with the receptor and it mainly interacts with the solvent. These low-frequency interactions take place primarily with L324, L144, Y325, and Q323.

The results of the docking of compound 7 to the deoxyuridine monophosphate binding site of TS in the presence of tetrahydrofolate shows a large network of interactions between the ligand and the receptor. The most frequent ones along the selected MD snapshots are with the cofactor, R29, E68, W90, Y116, L173, C176, H177, D216, N224, H254, and Y256. Furthermore, the carbonyl and hydroxyl groups of the ligand can hydrogen bond the receptor in more than 75% of the selected complex conformations. Despite the enoate moiety of the ligand makes more contact with TS than to QR, it is also located at the mouth of the binding site and exposed to the solvent. In contrast, the phenyl ring locates close to Y116 in a position that favors the *π*-*π* stacking of both rings.

Finally, compound 7 is predicted to bind to ST-PK in its ATP binding grove, making contact mainly with L436, V444, A457, K459, E474, I491, F507, I509, L510, L561, I573, and D574. Similarly to the predicted binding mode to TS, the complex shows that both the hydroxyl and carbonyl groups of the ligand are able to form hydrogen bonds with the receptor in most of the studied MD snapshots. Specifically, the carbonyl moiety accepts a hydrogen bond from the backbone of L510, while the hydroxyl substituent is able to hydrogen bond the side chains of K459 and E474. This predicted mechanism of action is in agreement with that previously proposed [52], in which the ligand interacts with K459, E474, and D574, a feature common to potent kinase inhibitors [53].

Congeneric series of compounds such as those presented in this research should follow the similarity principle, that is, similar compounds should have similar bioactivity profiles. To further assess the potential targets of the studied series of compounds we performed the same calculations carried out with compound 7 for chemicals 2 and 22. These are the ones with the higher potency after compound 7. The full statistics of their modeling processes are provided as Supporting Information. Interestingly, compounds 2 and 22 follow the same pattern of predicted targets as compound 7. Specifically, compound 2 is predicted to bind stably to the TS and ST-PK proteins while its *Δ*G of binding predicts an unstable complex with TS. On the other hand, compound 22 is predicted to form stable complexes with the same three potential targets predicted for compound 7: TS, QR, and ST-PK. In addition, unfeasible complexes are predicted between compounds 2 and 22 and the L-14a-D, C-8-SI, PO, and CA receptors as for compound 7.

**Figure 3:**
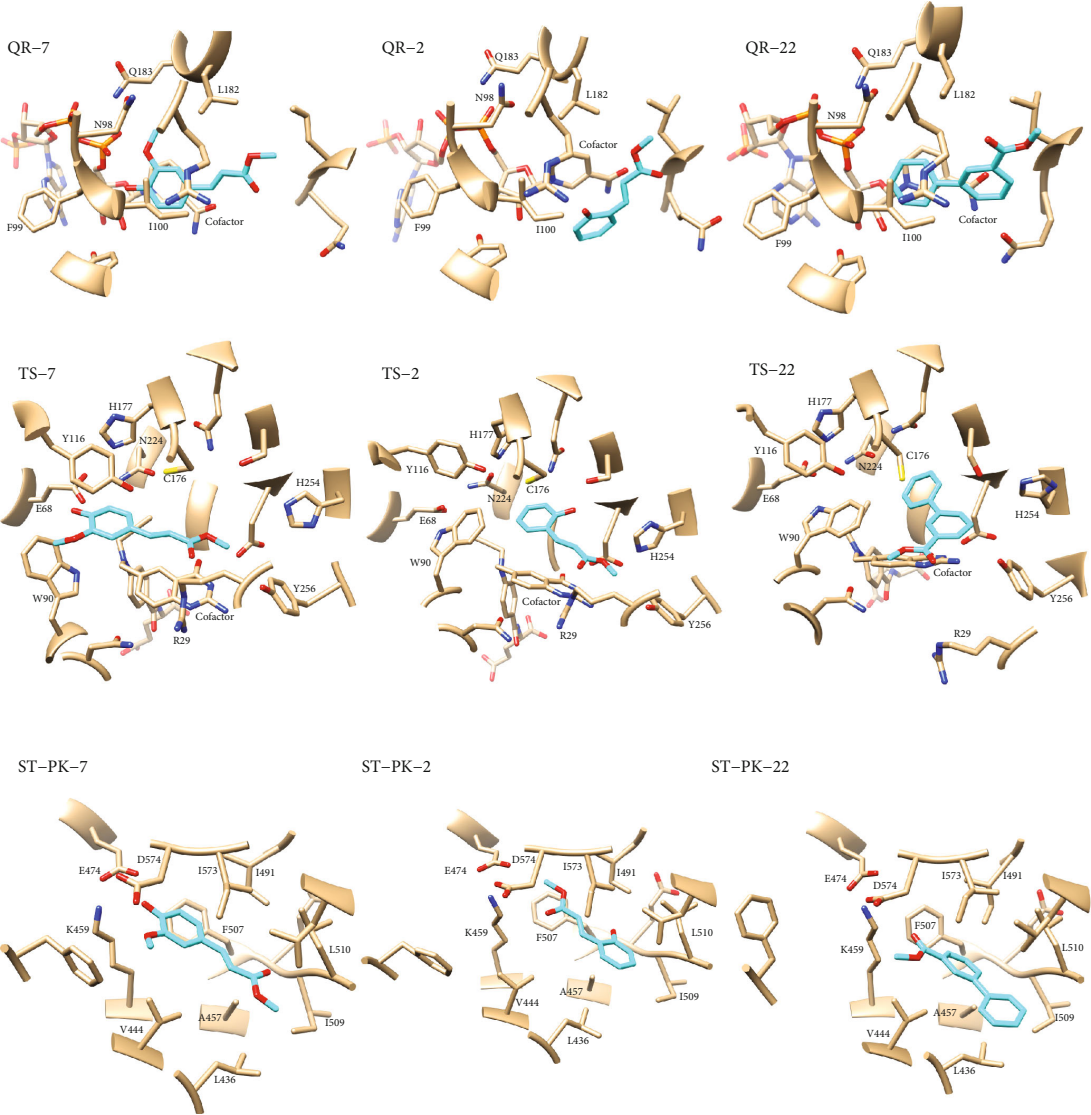
Predicted binding modes of compounds 7, 2, and 22 to QR, TS, and ST-PK of *Candida albicans*. Compounds are depicted cyan and the receptors in gray, with noncarbon atoms following the scheme: blue for N, red for O, yellow for S, and orange for P. Only residues interacting with the ligand in more than 50% of the analyzed MD snapshots are labelled in the complexes' structures. The complexes with each receptor were superimposed before generating the images.

The most stable complexes formed by compounds 7, 2, and 22 (see Table 3 and Supporting Information) with TS, QR, and ST-PK are given in Figure 3. One common feature among the complexes predicted for compound 7 with TS, QR, and ST-PK is the presence of hydrogen bonds to the receptor mediated by the p-hydroxyl substituent at its phenyl ring. The importance of these interactions is highlighted by the analysis of the predicted binding modes of compounds 2 and 22 to the same targets. The lack of this group in the later makes these interactions impossible, thus changing in different degrees the geometry of the predicted ligand-receptor complexes. The importance of these hydrogen bonds can be illustrated by the predicted binding modes of compounds 2 and 22 to ST-PK. These compounds are rotated 180° relative to the predicted orientation of compound 7 on ST-PK. This rotation restores the hydrogen bond interactions of these ligands with K459, E474, and D574 which are predicted to stabilize the complex with compound 7. Interestingly, such an orientation is impossible for compound 7 since its bulky p-methoxy substituent would clash with ST-PK residues I491 and E508.

Another interesting observation is that the removal of the p-hydroxyl and m-methoxy groups in compounds 2 and 22 reduces the volume of the cavity occupied by these ligands at the bottom of the binding site of TS. In consequence, W90 is predicted to rotate its side chain toward the binding pocket to occupy the space filled by these substituents in compound 7. This large rotation of W90 reduces the volume of the TS binding cavity and still allows for favorable ligand-receptor interactions with compounds 2 and 22.

QR is a flavodoxin-like protein that is involved in the control of oxidative stress in *Candida albicans* [54]. In consequence, the inhibition of this enzyme could induce oxidative stress in this fungus, ultimately leading to cell death. In a similar way, TS is a validated target against *Candida albicans.* There is interference in DNA synthesis and repair if it is inhibited [55]. In addition, ST-PK has been shown to be involved in the hyphal growth in *Candida albicans* [56], and it has been proposed as an effective target for *Candida albicans* inhibition.

The proposed mechanisms of action are in agreement with the observed structure-activity relationship for the assayed series of compounds. For example, the lack of the enoate moiety present in compound 7 (see compound 18) reduces the number of favorable contacts with all receptors. More important, the removal of this moiety abolishes or reduces the number or predicted hydrogen bonds between the most active compounds and the receptors. In the same vein, the presence of the hydroxyl substitution in the phenyl ring allows for the formation of hydrogen bonds with functionally important residues in all cases. This is exemplified by the loss of activity observed for compound 9. Besides the positive influence of a hydroxyl group attached to the phenyl ring, the addition of a second methoxy substituent in the phenyl ring leads to a reduction in the bioactivity of the studied compounds. The latter can be explained by the steric constraints that this substitution imposes as the addition of a second methoxy group contributes with steric clashes with the receptors (see compounds 7, 8, and 9).

## 4. Conclusions

In summary, we have prepared a set of derivatives of benzoic and cinnamic acids and investigated for their antifungal activity against three *Candida* strains. Additionally, a preliminary Structure-Activity Relationship study also was performed, permitting the recognition of some important structural requirements for antifungal activity and providing information for a better optimization of these classes of compounds as potential antimycotic agents. Molecular docking and the estimation of the free energies of binding were also attributed to the most potent compounds against *Candida* strains: 7, 2, and 22. The results show that the antifungal activity of these compounds could be due to its binding on multiple targets and have suggested the contribution of substituent groups to bioactivity.

## Figures and Tables

**Table 1:** Minimal inhibitory concentrations (MICs) values for compounds 1-23.

Compounds	MIC (*μ*g/ml)		
	*C. albicans* (ATCC® 10231™)	*C. parapsilosis* (ATCC® 22019™)	*C. krusei* (ATCC® 6258™)
1	250	250	250
2	62.5	62.5	125
3	250	250	250
4	250	125	125
5	250	250	250
6	250	250	250
7	62.5	31.25	62.5
8	250	125	125
9	500	250	250
10	250	250	250
11	500	250	500
12	500	500	500
13	250	250	250
14	500	250	500
15	500	250	250
16	500	500	500
17	250	125	125
18	500	250	250
19	250	250	250
20	250	250	500
21	250	250	250
22	62.5	62.5	62.5
23	500	500	500

**Table 2:** Summary of the docking of compound 7 to its potential targets.

Target	Conformer	CHEMPLP		GoldScore		ChemScore		ASP		Consensus *Z*-score
		Score	*Z*-score	Score	*Z*-score	Score	*Z*-score	Score	*Z*-score	
L-14a-D	1	57.54	2.50	27.21	3.21	22.96	0.38	29.54	1.07	1.79
	2	57.48	2.48	16.56	-0.65	27.95	2.19	30.58	1.43	1.36

C-8-SI	1	41.59	2.47	19.52	1.10	17.84	1.70	23.81	0.53	1.45
	2	39.73	1.63	19.58	1.13	16.54	1.00	24.04	0.66	1.11
	3	35.42	-0.31	21.05	1.76	16.46	0.96	26.24	1.90	1.08

TS	1	39.90	3.70	35.31	3.59	9.06	0.06	21.88	0.82	2.04
	2	34.46	1.46	22.10	1.01	11.61	2.14	22.60	1.05	1.41

PO	1	55.61	2.02	35.42	2.55	20.19	1.47	38.76	1.34	1.85
	2	55.82	2.09	30.74	1.22	18.19	0.66	39.43	1.51	1.37

QR	1	36.26	0.33	25.83	1.60	15.48	0.15	21.28	2.56	1.16
	2	36.72	0.49	24.02	1.25	20.01	2.69	16.54	-0.10	1.08

ST-PK	1	57.16	3.64	31.30	3.12	22.65	2.77	26.88	2.59	3.03

CA	1	45.04	2.71	32.49	0.34	14.39	0.32	17.80	0.28	0.91

**Table 3:** Predicted free energies of binding of compound 7 to its potential targets. Values are expressed in kcal/mol.

Target	Conformer	MM-PBSA component							*Δ*G_Binding_
		VDWAALS	EEL	EPB	ENPOLAR	EDISPER	DELTA G gas	DELTA G solv	
L-14a-D	1	-29.42	-57.64	143.64	-23.72	38.89	-87.06	158.80	71.74
	2	-24.49	-64.23	148.01	-23.35	39.55	-88.71	164.21	75.50

C-8-SI	1	-31.14	-10.55	79.46	-23.39	40.21	-41.69	96.27	54.58
	2	-31.36	-22.75	74.72	-23.49	39.02	-54.11	90.26	36.14
	3	-30.21	-17.25	63.11	-22.58	38.94	-47.46	79.47	32.01

TS	1	-34.44	-14.93	40.06	-23.27	40.34	-49.37	57.13	7.76
	2	-33.58	-27.34	40.52	-23.58	39.87	-60.92	56.81	-4.11

PO	1	-34.84	-26.71	57.87	-23.19	41.09	-61.54	75.77	14.22
	2	-33.13	-17.74	44.26	-23.02	40.48	-50.87	61.71	10.84

QR	1	-28.78	-10.90	26.61	-21.06	34.61	-39.69	40.16	0.47
	2	-30.48	-17.08	30.38	-20.75	33.98	-47.56	43.61	-3.96

ST-PK	1	-28.99	-24.94	33.55	-22.70	36.52	-53.93	47.37	-6.56

CA	1	-14.72	-32.02	38.76	-14.37	24.75	-46.74	49.14	2.39

## Data Availability

Part of the article data used to support the findings of this study has been deposited in the Federal University of Paraíba repository at https://repositorio.ufpb.br/jspui/handle/123456789/1356.

## Supplementary Materials

Supplementary Materials Predicted compound 7–L-14a-D complex. Predicted compound 7–C-8-SI complex. Predicted compound 7–PO complex. Predicted compound 7–CA complex. Docking results for compound 2. Docking results for compound 22 MM-PBSA results for compound 2 MM-PBSA results for compound 22.
