# Supplementary material for: Bioactivity and Molecular Docking Studies of Derivatives from Cinnamic and Benzoic Acids
Source: Biomed Res Int. 2020 May 21;2020:6345429. doi: 10.1155/2020/6345429 (PMC7273447; doi:10.1155/2020/6345429)
Supplement: Supplementary Materials — Predicted compound 7–L-14a-D complex. Predicted compound 7–C-8-SI complex. Predicted compound 7–PO complex. Predicted compound 7–CA complex. Docking results for compound 2. Docking results for compound 22 MM-PBSA results for compound 2 MM-PBSA results for compound 22. [file 6345429.f1.pdf]

Supporting Information for article

Bioactivity and Molecular Docking Studies of Derivatives from Cinnamic and Benzoic Acids

Yunierkis Perez-Castillo,[1] Tamires C. Lima,[2] Alana R. Ferreira,[3] Cecília R. Silva,[4] Rosana S. Campos,[4] João B. A. Neto,[4] Hemerson I. F. Magalhães,[3] Bruno C. Cavalcanti,[5] Hélio V. N. Júnior,[4] and Damião P. de Sousa[3]

[1] Escuela de Ciencias Físicas y Matemáticas, Universidad de Las Américas, Quito, Ecuador
[2] Department of Pharmacy, Federal University of Sergipe, CEP 49100-000, São Cristóvão, Sergipe, Brazil
[3] Department of Pharmaceutical Sciences, Federal University of Paraíba, CEP 58051-970, João Pessoa, Paraíba, Brazil
[4] Department of Clinical and Toxicological Analysis, School of Pharmacy, Laboratory for Bioprospection and Experiments in Yeast, LABEL, Federal University of Ceará, Fortaleza, CE, Brazil
[5] Department of Physiology and Pharmacology, Federal University of Ceará, Fortaleza, CE, Brazil

Correspondence should be addressed to Damião P. de Sousa; damiao_desousa@yahoo.com.br

Contents

Predicted compound 7 – L-14a-D complex 2
Predicted compound 7 – C-8-SI complex 3
Predicted compound 7 – PO complex 4
Predicted compound 7 – CA complex 5
Docking results for compound 2 6
Docking results for compound 22 7
MM-PBSA results for compound 2 8
MM-PBSA results for compound 22 9

Predicted compound 7 – L-14a-D complex

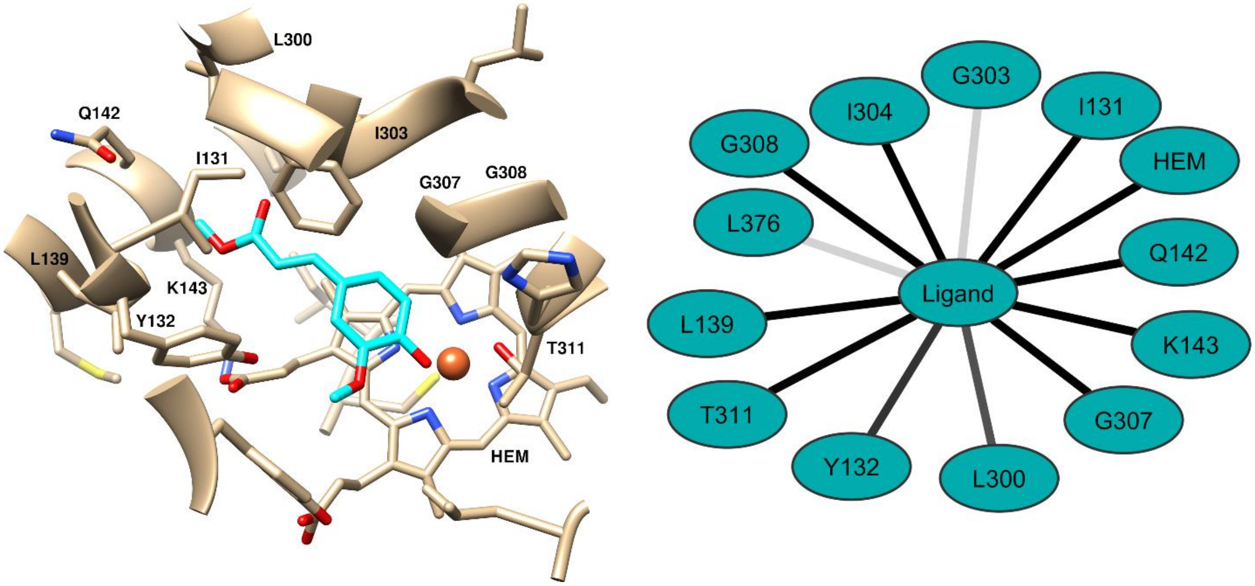

*Figure S1 Predicted binding mode of compound **7** to C. albicans L-14a-D. Compound **7** is depicted cyan and the receptor in gray, with non-carbon atoms following the scheme: blue for N, red for O, yellow for S and orange for P. On the right side of the figure are represented the predicted interaction frequencies with the residues at the receptor binding site. Darker lines indicate the higher frequencies of interaction. Only residues interacting with the ligand in more than 50% of the analyzed MD snapshots are labelled in the complex structures.*

Compound **7** binds to L-14a-D with its hydroxyl group pointing to the iron ion of the receptor's heme group located at the bottom of the binding pocket and with its phenyl ring stacking in front of the same cofactor. Most of the interactions observed in this predicted complex are of Van der Waals type. The ligand is observed to interact with I131, Y132, L139, Q142, K143, L300, G303, I304, G307 and T311 in the majority of the analyzed MD snapshots. Hydrogen bonds are predicted to occasionally form between the ligand's hydroxyl substituent and either the side chain of T311 or the backbone of G307. These hydrogen bonds are predicted in 26% of the studied complex conformations.

Predicted compound 7 – C-8-SI complex

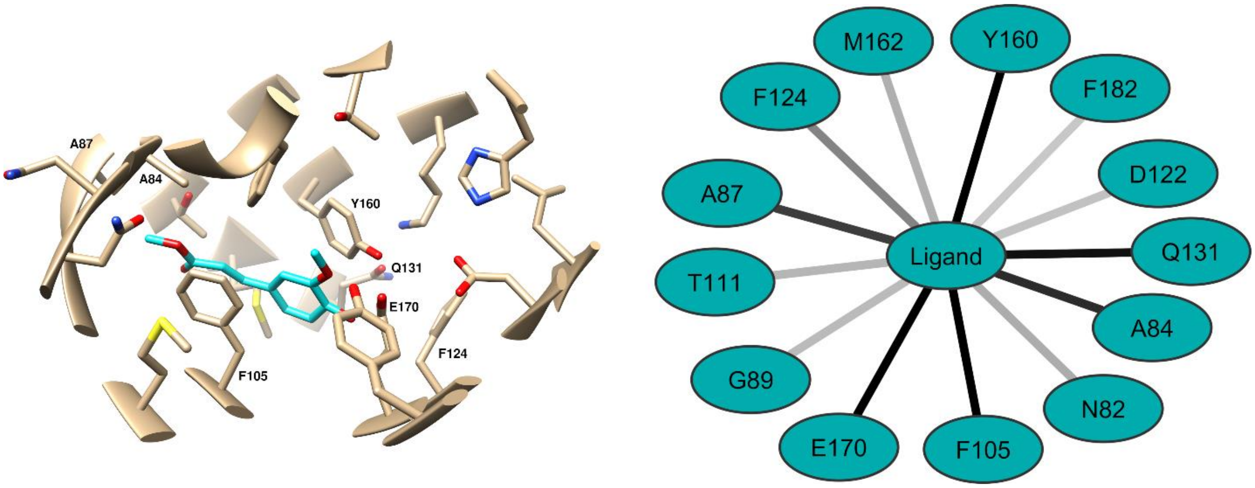

*Figure S2 Predicted binding mode of compound **7** to C. albicans C-8-SI. Compound **7** is depicted cyan and the receptor in gray, with non-carbon atoms following the scheme: blue for N, red for O, yellow for S and orange for P. On the right side of the figure are represented the predicted interaction frequencies with the residues at the receptor binding site. Darker lines indicate the higher frequencies of interaction. Only residues interacting with the ligand in more than 50% of the analyzed MD snapshots are labelled in the complex structures.*

The predicted binding mode of compound **7** to C-8-SI shows that its phenyl ring stacks in front of Y160. In more than 50% of the analyzed MD snapshots the hydroxyl substituent hydrogen bonds to the side chains of D122, Q131 and E170. A less frequent hydrogen bond (in less than 20% of the analyzed MD snapshots) is predicted between the carbonyl group of compound **7** and the side chain of T111. In addition, the most frequent interactions of the ligand with the receptor take place with A84, A87, F105, F124, Q131, Y160 and E170.

Predicted compound 7 – PO complex

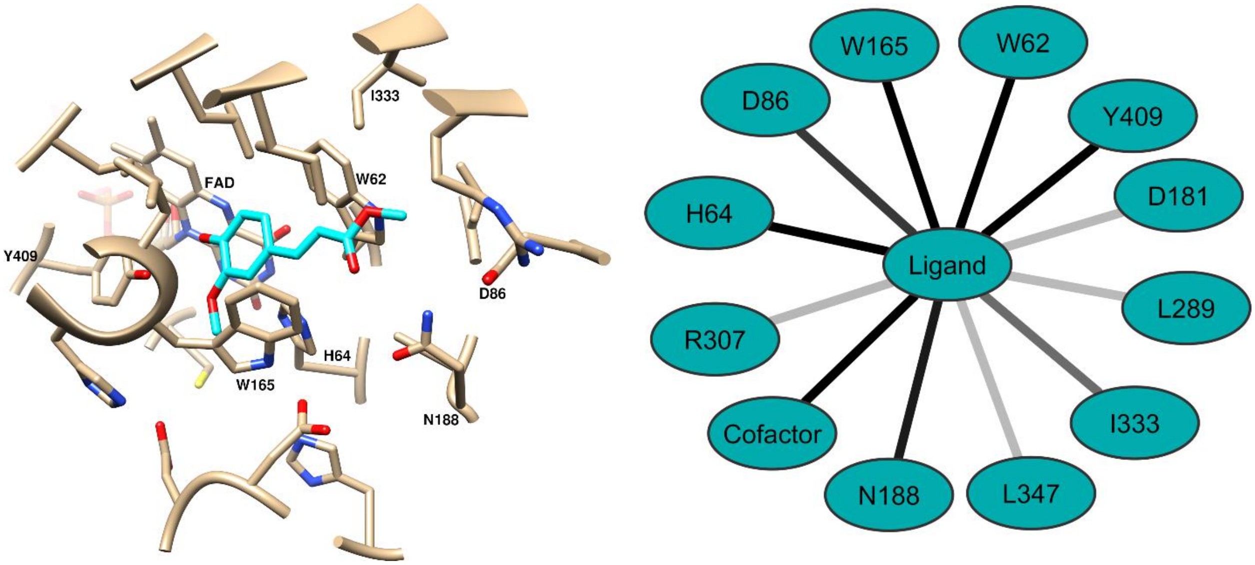

*Figure S3 Predicted binding mode of compound **7** to C. albicans PO. Compound **7** is depicted cyan and the receptor in gray, with non-carbon atoms following the scheme: blue for N, red for O, yellow for S and orange for P. On the right side of the figure are represented the predicted interaction frequencies with the residues at the receptor binding site. Darker lines indicate the higher frequencies of interaction. Only residues interacting with the ligand in more than 50% of the analyzed MD snapshots are labelled in the complex structures.*

The predicted binding mode of compound **7** to PO presents the substituted phenyl ring oriented toward the bottom of the binding pocket, facing the FAD cofactor of the enzyme. On the other hand, the eonate substituent points to the entrance of the binding cavity. The residues interacting more frequently with the ligand are the FAD cofactor, W62, H64, D66, W165, N188, I333 and Y409. The aromatic moiety of compound **7** occupies the space between the cofactor, W62, H64 and W165. However, its orientation does not favor the π-π stacking of the ligand with any of the later amino acids. Furthermore, the compound is predicted to hydrogen bond the side chains of N188 and Y409 through its carbonyl and hydroxyl substituents, respectively. Among these, the hydrogen bond with N188 is observed in 64% of the MD snapshots employed for MM-PBSA calculations while that predicted with Y409 is observed in 32% of them.

Predicted compound 7 – CA complex

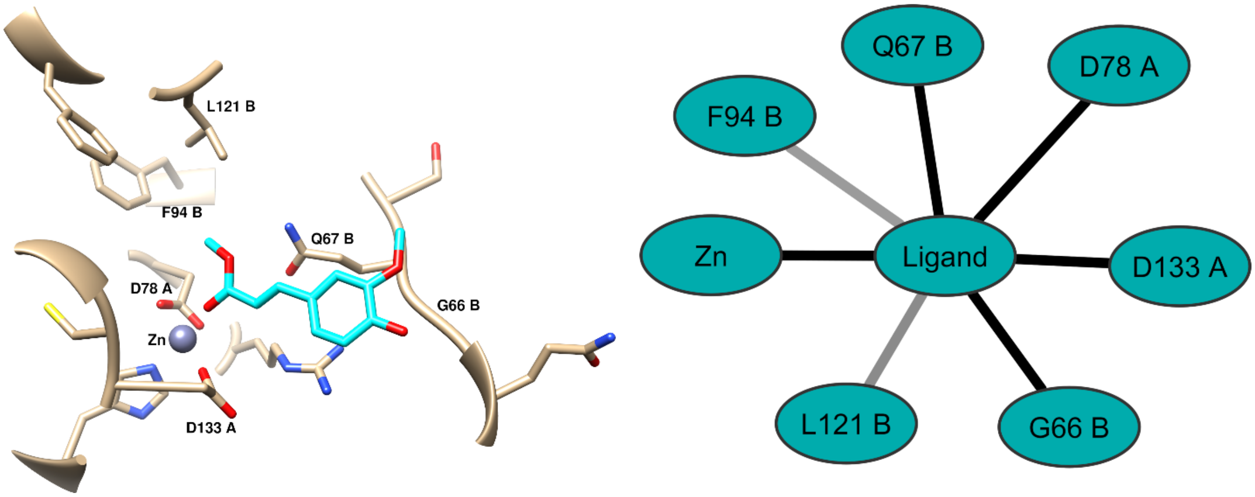

*Figure S4 Predicted binding mode of compound **7** to C. albicans CA. Compound **7** is depicted cyan and the receptor in gray, with non-carbon atoms following the scheme: blue for N, red for O, yellow for S and orange for P. On the right side of the figure are represented the predicted interaction frequencies with the residues at the receptor binding site. Darker lines indicate the higher frequencies of interaction. Only residues interacting with the ligand in more than 50% of the analyzed MD snapshots are labelled in the complex structures.*

Compound **7** orients with its carbonyl group pointing to the Zn^{2+} ion of CA, while the phenyl substituted moiety is located at the entrance of the binding pocket. In addition, it interacts with a few residues from the two chains that form the CA homodimer in more than 100 out of the 200 analyzed MD snapshots. These residues are D78 and D133 of the chain A as well as G66, Q67, F94 and L121 from the B subunit. No hydrogen bond is predicted to form between the ligand and the receptor.

Docking results for compound 2

Target	Conformer	CHEMPLP		GoldScore		ChemScore		ASP		Consensus Z-Score
		Score	Z-Score	Score	Z-Score	Score	Z-Score	Score	Z-Score	
L-14a-D	1	57.54	2.50	27.21	3.21	22.96	0.38	29.54	1.07	1.79
	2	57.48	2.48	16.56	-0.65	27.95	2.19	30.58	1.43	1.36
	3	49.96	0.81	19.17	0.30	28.41	2.36	28.23	0.62	1.02
C-8-SI	1	42.21	2.19	24.45	2.23	15.51	-0.14	22.38	1.03	1.33
	2	37.62	0.12	20.04	0.69	17.69	1.07	25.81	2.63	1.13
	3	41.76	1.98	21.53	1.21	15.53	-0.13	22.51	1.10	1.04
TS	1	39.78	2.69	31.07	1.94	14.35	2.47	23.91	1.42	2.13
	2	40.56	2.95	33.21	2.35	10.53	0.46	24.82	1.70	1.87
PO	1	53.69	2.37	30.49	1.88	19.50	0.64	34.17	0.98	1.47
	2	50.98	1.40	26.65	0.29	22.36	1.82	35.25	1.36	1.22
	3	49.68	0.94	29.57	1.50	21.21	1.34	33.40	0.71	1.12
QR	1	39.86	1.42	24.86	0.91	16.72	0.58	21.87	2.32	1.31
	2	39.99	1.47	27.95	1.58	16.82	0.62	19.20	1.16	1.21
ST-PK	1	47.94	1.69	22.68	1.35	20.62	1.28	24.08	2.81	1.78
	2	48.37	1.82	22.99	1.45	20.71	1.34	19.46	-0.30	1.08
	3	46.17	1.16	22.00	1.12	20.75	1.37	20.80	0.60	1.06
CA	1	40.75	1.43	32.59	1.93	21.48	3.72	15.73	0.99	2.02
	2	47.14	3.27	33.96	2.13	13.95	0.05	16.08	1.09	1.63

Docking results for compound 22

Target	Conformer	CHEMPLP		GoldScore		ChemScore		ASP		Consensus Z-Score
		Score	Z-Score	Score	Z-Score	Score	Z-Score	Score	Z-Score	
L-14a-D	1	71.88	3.09	35.08	1.58	33.08	2.68	26.41	-1.22	1.53
	2	64.58	1.69	30.13	0.43	30.19	1.03	33.51	2.07	1.30
	3	63.58	1.49	27.22	-0.25	33.31	2.81	30.03	0.46	1.13
C-8-SI	1	42.21	2.19	24.45	2.23	15.51	-0.14	22.38	1.03	1.33
	2	37.62	0.12	20.04	0.69	17.69	1.07	25.81	2.63	1.13
TS	1	45.13	2.71	38.53	2.74	16.04	1.72	25.22	1.40	2.14
	2	47.21	3.33	31.26	1.43	15.47	1.28	26.35	1.70	1.94
	3	39.72	1.08	30.30	1.26	16.16	1.81	23.89	1.05	1.30
PO	1	62.04	1.58	41.74	1.82	25.26	1.13	39.73	1.17	1.43
	2	62.52	1.69	38.82	1.18	24.22	0.83	40.81	1.40	1.27
	3	62.47	1.68	36.67	0.70	26.38	1.46	38.92	1.00	1.21
QR	1	50.29	1.66	37.00	1.72	24.94	1.87	28.04	2.12	1.84
	2	49.12	1.31	37.97	1.89	24.13	1.29	24.51	0.91	1.35
	3	50.43	1.70	28.43	0.22	24.83	1.79	24.19	0.80	1.13
ST-PK	1	55.86	1.80	29.03	1.64	26.82	1.62	29.39	2.56	1.91
	2	58.13	2.50	27.09	0.89	28.23	2.68	25.60	0.93	1.75
	3	57.01	2.15	25.59	0.32	27.81	2.36	24.88	0.62	1.36
CA	1	45.97	1.97	22.03	0.04	22.03	2.54	16.13	0.26	1.20

MM-PBSA results for compound 2

Target	Conformer	MM-PBSA Component							DELTA TOTAL
		VDWAALS	EEL	EPB	ENPOLAR	EDISPER	DELTA G gas	DELTA G solv	
L-14a-D	1	-18.09	-82.51	144.98	-20.34	34.32	-100.60	158.96	58.36
	2	-23.53	-50.95	107.98	-21.06	35.04	-74.48	121.96	47.48
	3	-29.51	-19.01	45.82	-21.60	35.26	-48.52	59.48	10.97
C-8-SI	1	-28.86	-16.51	51.38	-20.69	34.12	-45.37	64.81	19.44
	2	-25.98	-3.65	45.24	-19.98	34.70	-29.64	59.96	30.32
	3	-29.24	0.03	65.96	-20.87	34.92	-29.21	80.01	50.80
TS	1	-29.48	-17.19	28.08	-20.41	33.28	-46.67	40.95	-5.72
	2	-29.20	-22.05	40.95	-20.63	34.23	-51.25	54.56	3.31
PO	1	-31.77	-21.64	51.30	-20.97	35.43	-53.41	65.77	12.36
	2	-30.44	-1.18	40.63	-20.60	35.96	-31.63	55.98	24.36
	3	-29.05	-24.00	48.30	-20.49	35.41	-53.05	63.23	10.18
QR	1	-24.63	-11.58	28.63	-18.18	30.04	-36.22	40.49	4.27
	2	-23.94	-7.34	23.27	-17.62	29.02	-31.28	34.67	3.39
ST-PK	1	-22.28	-5.71	19.72	-18.06	30.52	-27.99	32.18	4.19
	2	-25.84	-13.07	24.66	-19.97	31.69	-38.91	36.38	-2.53
	3	-27.76	-15.41	25.84	-20.78	32.08	-43.16	37.13	-6.03
CA	1	-26.41	-12.05	34.84	-20.02	30.93	-38.46	45.74	7.28
	2	-15.28	-27.29	42.28	-13.85	23.12	-42.56	51.55	8.99

MM-PBSA results for compound 22

Target	Conformer	MM-PBSA Component							DELTA TOTAL
		VDWAALS	EEL	EPB	ENPOLAR	EDISPER	DELTA G gas	DELTA G solv	
L-14a-D	1	-24.80	-74.91	130.98	-24.62	41.17	-99.71	147.52	47.81
	2	-33.61	-10.34	32.60	-24.68	41.07	-43.95	48.98	5.04
	3	-23.56	-73.11	100.96	-25.07	39.68	-96.67	115.57	18.90
C-8-SI	1	-34.55	5.50	60.80	-24.02	40.24	-29.06	77.02	47.97
	2	-33.44	12.81	96.12	-24.08	42.13	-20.63	114.18	93.55
TS	1	-31.11	-10.55	24.62	-21.81	37.09	-41.66	39.90	-1.76
	2	-36.65	-13.11	34.27	-24.63	41.08	-49.76	50.72	0.96
	3	-33.82	-8.22	33.45	-23.33	40.86	-42.04	50.98	8.94
PO	1	-35.73	-18.20	53.52	-23.51	42.25	-53.93	72.26	18.33
	2	-36.73	-10.95	38.69	-23.92	42.14	-47.68	56.91	9.23
	3	-34.08	-20.20	46.40	-23.05	41.14	-54.28	64.48	10.20
QR	1	-31.42	-7.47	25.21	-22.30	37.01	-38.88	39.92	1.04
	2	-29.50	-5.58	21.20	-20.77	34.30	-35.08	34.72	-0.36
	3	-29.00	-8.56	24.31	-20.64	34.41	-37.56	38.08	0.53
ST-PK	1	-28.65	-10.25	20.97	-22.24	36.88	-38.90	35.61	-3.29
	2	-28.89	-4.87	17.36	-22.07	36.44	-33.76	31.73	-2.03
	3	-30.03	-2.69	16.30	-22.61	36.72	-32.72	30.40	-2.32
CA	1	-28.24	-5.71	23.65	-21.82	34.14	-33.95	35.96	2.01
